# Heart rate variability measurement and influencing factors: Towards the standardization of methodology

**DOI:** 10.21542/gcsp.2024.35

**Published:** 2024-08-01

**Authors:** Narjisse Damoun, Youssra Amekran, Nora Taiek, Abdelkader Jalil El Hangouche

**Affiliations:** Department of Physiology, Faculty of Medicine and Pharmacy of Tangier, Abdelmalek Essaadi University, Tangier, Morocco

## Abstract

Heart rate variability (HRV) is widely recognized as an effective and valuable tool for evaluating cardiac autonomic modulation. However, various factors can influence HRV before and during assessment, complicating the interpretation and comparability of results. This review outlines the different factors affecting HRV and underscores the importance of considering them to ensure consistent and reliable HRV outcomes. Key influencing factors are categorized into physiological (e.g., age, gender, genetics), lifestyle (e.g., physical activity, alcohol use, smoking, drugs, diet), environmental (e.g., time of day, temperature, noise), and methodological (e.g., body position, recording duration, and respiration) domains. Knowing these factors can help researchers and physicians gain a better understanding of HRV and improve the interpretation of their findings. Consequently, this can lead to the development of standardized methods for consistently assessing and interpreting HRV measures in clinical practice.

## Introduction

The autonomic nervous system (ANS), which comprises the sympathetic and parasympathetic nervous systems, regulates various functions of the body. In conjunction with endocrine and immunological systems, it maintains body homeostasis by adjusting physiological activity in response to disturbances from both the internal and external environments^1^.

The ANS is a complex neural network that is evaluated through a variety of tests^2^. While invasive measurements are considered the most accurate, they are limited by their nature and inability to be performed frequently^3^. However, Ewing and Clarke introduced a non-invasive battery of tests aimed at evaluating autonomic function through the stimulation of cardiovascular reflexes. These tests have been widely employed in the clinical assessment of cardiovascular autonomic neuropathy^4^.

Ewing’s battery includes parasympathetic function tests (HR response to standing from the supine posture (30:15 ratio), Valsalva maneuver, and slow deep breathing), while sympathetic function was assessed using the sustained handgrip and active standing from the supine posture tests^2^. Clinicians are encouraged to administer the complete Ewing battery of tests to ensure the reliability of the results, although this may require significant patient cooperation and time commitment, thereby potentially limiting their widespread utilization^5^.

Recently, heart rate variability (HRV) has emerged as a simple, non-invasive, and easily applicable tool for the diagnosis of autonomic dysfunction^5,6^. HRV is defined as a measure of the variation in time between consecutive normal-to-normal R-R intervals on the electrocardiogram (ECG)^7^. This variability is influenced by the interplay between the sympathetic and parasympathetic systems^1^. In general, HRV serves as a biomarker for health status. Lower HRV values suggest abnormal adaptation associated with ANS insufficiency, whereas higher values indicate effective adaptation and optimal functioning of autonomic mechanisms^6^.

HRV analysis is based on short-term (usually 5–20 min) or long-term (24-hour) ECG recordings using various parameters: time-domain, frequency-domain, and non-linear indices^8^.

The time-domain parameters of HRV reflect the time period between successive heartbeats and are calculated from normal-to-normal intervals^9^. The most used time-domain parameters are: the standard deviation of normal-to-normal intervals (SDNN), reflecting the overall variation within the RR interval series, the root mean square of successive differences (RMSSD), and the proportion of adjacent RR intervals differing by more than 50 ms (pNN50), reflecting parasympathetic modulation of the heart^9,10^.

The frequency domain analysis enables the measurement of HRV by decomposing signals into frequency bands linked to various components of the ANS^11^. The European Society of Cardiology and the North American Society for Pacing and Electrophysiology categorized HRV into four frequency bands: ultra-low frequency (ULF) ( ≤ 0.003 Hz), very low frequency (VLF) (0.003–0.04 Hz), low frequency (LF) (0.04–0.15 Hz) and high frequency (HF) (0.15–0.40 Hz)^9^. The HF band reflects parasympathetic modulation, while the LF band is an indicator of both the sympathetic and vagal tone^9,11^. Another parameter, the LF/HF ratio, has been suggested to assess sympathovagal balance. A low LF/HF ratio is indicative of parasympathetic dominance, whereas a high LF/HF ratio indicates sympathetic dominance^12^.

Finally, non-linear methods are thought to provide a better expression of the complex nature of HRV, and a more comprehensive description of the nonlinear interaction between the parasympathetic and sympathetic nervous system branches^10,13^. Non-linear methods include detrended fluctuation analysis (DFA), power law relationship analysis, approximate entropy, and Point Correlation Dimension (PD2i), which have demonstrated their ability to provide prognostic information^10^.

Despite the potential usefulness of HRV measurements in medical and research contexts, an ongoing debate persists regarding the methodology that accurately reflects meaningful outcomes^14^. One reason for this could be the lack of standardization in data collection methods and analytical techniques used during HRV assessment, which stems from the heterogeneity of measurement protocols and software tools availablility^11,15^. In addition, HRV is highly sensitive to various factors, some of which are unmodifiable, such as physiological, pathological and psychological conditioning^16^.

Other factors are related to the patient’s lifestyle, such as sleep quality, physical activity, alcohol consumption, and drug use^16,17^. Additionally, methodological challenges related to acquiring and analyzing heart rate data may also affect HRV^18^.

The Task Force of the European Society of Cardiology and the North American Society of Pacing and Electrophysiology have provided valuable information regarding the standard measurements and physiological significance of the HRV parameter^9^. Although recent publications have updated various aspects of Task Force guidelines, and several studies have proposed recommendations for the experimental planning, it is important to note that these recommendations do not encompass all factors impacting HRV measurement^11,19^. Consequently, physicians must maintain awareness and develop a comprehensive understanding of these factors to ensure accurate measurement, analysis, and interpretation^17,20^.

This review aims to provide a comprehensive and critical report of the factors influencing HRV measurement. It aims to highlight the current limitations and challenges within HRV research, including variability in methodologies, interpretation, and clinical application. Additionally, the review encourages additional research aimed at developing and standardizing HRV methodologies to ensure consistency and reliability in HRV assessment.

By addressing these issues, the review aspires to enhance the accuracy of HRV measures, thereby enabling physicians to better assess and interpret HRV in clinical settings. Ultimately, this will improve the clinical utility and application of HRV in monitoring health, and diagnosing conditions.

## Physiological factors

Before starting an HRV measurement, it is important to consider the different factors that can influence HRV. Among these factors remain physiological variables that cannot be controlled and represent a source of individual differences such as age, gender, and genetics.

It has been shown that HRV decreases with aging, which could be explained by the maturation of the ANS, which attenuates autonomic functions^21^. A study conducted within a healthy adult Asian population objectified, through regression analysis, that various HRV indices decline with age, as observed in the Western population^22^. It was shown that SDNN and SDANN parameters exhibit a linear decline as age increases, except the 50–59 year age bracket.

This linear association is reversed in individuals aged 60 years and above, where RMSSD and pNN50 parameters increase^23^. Frequency domain parameters, mainly HF and LF, have also been demonstrated to decrease with aging^24^. Moreover, significant decline of all 24-hour HRV parameters was demonstrated to occur between the second and third decades of life^25^. This decline in HRV seems to affect significantly more women than men^25,26^.

Additionally, this association between aging and HRV decline can lead to higher risk of mortality. Consequently, this might limit the predictivity of HRV, especially in the elderly, where distinguishing between HRV values arising from aging process and those arising from health conditions becomes challenging^27^.

Considering these findings, researchers should stratify their samples by age or use age-adjusted norms when analyzing HRV data. Longitudinal studies are particularly valuable for understanding how HRV changes over the lifespan.

Several studies have reported gender differences in HRV. According to a recent meta-analysis, females had a significantly lower mean R-R interval and SDNN values compared to males. Additionally, the power spectral density of HRV in females was characterized by significantly less total power, with an increased HF power and a decreased LF power^28^.

In another study, short-term and long-term HRV recordings revealed a predominance of parasympathetic tone over sympathetic tone in women, while the opposite was observed in men^24^. In an analysis of SDNN, SDANN, and PNN50 parameters, statistically significant sex-related differences were observed (with corresponding *p*-values of *P* < 0.001, *P* < 0.001, and *P* = 0.017, respectively), where males showed higher values compared to females^23^. In contrast, no significant gender differences were reported in other studies^29^.

Given the hormonal variations and physiological differences between males and females, gender should be considered as a potential modifying factor in HRV studies. Researchers might need to analyze data separately for males and females or incorporate gender as a covariate in their statistical models. Hence, clinicians should interpret HRV measures within the context of gender, as well as for gender.

Genetic factors have also been identified to affect HRV during either rest or stress conditions. These genetic characteristics are considered as one of the individual HRV disparities^17,26,30^. Notably, a study involving a sample of young adult twins revealed a significant heritability of HRV, implying that around 50–60% of the observed variability in HRV among individuals might be attributed to the influence of genetic components^31^.

## Lifestyle factors

### Physical activity

Numerous studies have shown that HRV is significantly affected by physical activity. After a 10-week exercise intervention, postmenopausal women had decreased SDNN, NN50, LF, HF, and LF/HF^32^. In elderly men, intensive interval training led to an increase in nocturnal parasympathetic HRV indices (PNN50, RMSSD, and HF)^33^. Individuals engaged in moderate and vigorous physical activity showed a higher HRV and lower HR^34^.

A study indicates that various exercise intervention modalities resulted in the improvement of HRV parameters among sedentary middle-aged adults^35^. In addition, it was highlighted that HRV tends to decrease as the exercise duration and intensity increase^36^. Therefore, a restriction of intense physical activity prior to HRV data collection is required^19^.

A quantitative analysis of post-exercise cardiac parasympathetic reactivation among athletes and healthy subjects was conducted considering exercise intensity, duration, and fitness/training status. The findings revealed that after a single aerobic training session, the total cardiac vagal reactivation can extend for up to 24 h following low-intensity aerobic exercise, 24–48 h after moderate-intensity exercise, and a minimum of 48 h after high-intensity exercise^37^.

Variability in physical activity levels among participants can significantly impact HRV outcomes. Researchers should standardize activity levels before data collection or account for them in their analysis. Studies may benefit from categorizing participants based on their fitness levels or including controlled exercise interventions to assess changes in HRV.

### Alcohol

The acute ingestion of alcohol leads to a reduction in HRV, which could be attributed to either sympathetic activation or parasympathetic inhibition^17,26,38^. A study revealed that the consumption of alcohol is associated with elevated LF normalized power and LF/HF ratio, along with a decrease in the total HRV and HF component. This consumption led to the activation of sympathetic outflow and a suppression of tonic vagal activity^39^.

Among non-alcohol-dependent individuals, the consumption of low doses is associated with elevated HRV parameters in comparison to those who drink less often or not at all. Conversely, a high alcohol consumption is associated with decreased HRV in both groups^40^.

Other results have demonstrated that habitual moderate alcohol drinkers exhibit higher HRV compared to nonhabitual drinkers^41^. It was noted that alcohol consumption’s effect on HRV is reversible if healthier lifestyle changes are implemented^17,21,26^. In terms of HRV measurement, it is recommended to avoid alcohol consumption 24 h prior to the assessment^19,42^.

Overall, discrepancies in the alcohol induced effects on HRV could be attributed to various factors. In general, the complexity of the interactions between alcohol consumption and autonomic function, coupled with the diverse study populations (e.g., patterns of alcohol intake, health status, tolerance to alcohol) and methodologies employed, likely underlie the discrepancies observed in the effects of alcohol on HRV across different studies.

### Smoking

Smoking has been found to reduce HRV. The parasympathetic manoeuvre has shown a decrease in HRV among heavy smokers, serving as an indicator of reduced vagal activity^43^. Nicotine, the primary constituent of tobacco, negatively impacts HRV in non-smoking adolescents when ingested orally. This results in an increase in LF and a decrease in HF, shifting cardiac autonomic modulation towards sympathetic predominance, as indicated by an elevated LF/HF ratio^44^.

A decrease in HRV has been observed in individuals with significant exposure to environmental tobacco smoke, indicating its adverse impact on cardiac autonomic function^45^. Nevertheless, studies suggest that tobacco’s effect on HRV can be reversed^21,26^. The period of smoking cessation prior to HRV measurement varies across studies. There are studies suggesting 10 h of tobacco restriction before starting the measurements and others 12 h^24,46,47^.

### Drugs

When interpreting HRV results, it is crucial to consider the influence of different drugs on HRV. This impact can vary significantly depending on the type of medication used and the characteristics of the population being studied^26^.

Numerous studies have examined the impact of various medications on HRV, contributing to a deeper understanding of how these substances can influence HRV. For example, beta-adrenergic blockers, such as Atenolol, metoprolol, and acebutolol have demonstrated their ability to decrease sympathetic activity in patients with cardiovascular diseases^38^. These medications play a role in restoring the balance between sympathetic and parasympathetic activity in cardiovascular disease^38^.

A systematic review has shown that among various psychotropic medications, only tricyclic antidepressants and clozapine demonstrated effects on HRV^48^. In other contexts, it has been shown that the intake of oral contraceptives by females may not affect HRV at rest, in contrast to stress conditions^49,50^. In conclusion, drugs are considered as one of the main influencing factors impacting HRV values^20^. Therefore, it is important when interpreting HRV to mention any cardiovascular-related medication administrated by the subjects to control for these factors^19^. Studies should carefully consider the inclusion or exclusion criteria regarding health conditions and medication use that might confound HRV data.

Given that certain diseases and medication could alter (inflate or deflate) the HRV values, HRV results must be interpreted in the context of the patient’s overall health and medication regimen.

### Caffeine

Studies have suggested that coffee consumption may impact HRV, leading to an enhancement in HF parameter of HRV. This effect could indicate an elevated parasympathetic activity^51^. Caffeine causes an acute increase in sympathetic nerve activity, blood pressure, catecholamine concentration, and plasma renin activity^51^. To ensure accurate HRV measurements, it is advised to avoid caffeine intake, 2 h prior to the measurements^19^.

### Food

Digestion may have an impact on HRV parameters, especially for short-term HRV measurements. Therefore, it is important to consider and establish a standardized timing for the last meal, particularly in investigations involving multiple HRV measurements^52^. This may help ensuring the reliability of data by mitigating potential interferences from food consumption. Temporary short-term food deprivation could influence autonomic bodily signals, which is reported to be associated with decreased sympathetic efference and altered vagal activity^53^.

Accordingly, an increase in HRV has been detected in children after food deprivation, particularly in the LF component^54^. In fasting pre-adolescents, an increase in LF and HF parameters was reported, whereas, in participants who had eaten, there were non-significant changes in both the HRV indices^55^.

In contrast, other studies found reduced HRV and increased HR after eating^53^. A study conducted by Ching-liang lu et al. showed that following a meal, the sympathetic and parasympathetic ratio remained elevated for up to one hour, indicating a decrease in vagal activity^56^. In this context, it was suggested to have a 2-hours period of food deprivation before measuring HRV^19^.

Based on these data, it becomes clear that controlling for dietary data is crucial. Experimental studies might standardize diet and abstain from some substances discussed in this review (such as caffeine and alcohol) prior to HRV measurement. In clinical settings, clinicians should consider dietary and lifestyle factors when evaluating HRV. Abrupt changes in HRV might be related to recent dietary changes or substance use, which could impact HRV interpretation.

### Sleep

The American Academy of Sleep Medicine recommends 7 to 9 h of sleep per day for adults^57^. Sleep deprivation has detrimental effects on neurocognitive functions and the cardiovascular autonomic system^58^. The relationship between sleep deprivation and the ANS is a subject of controversy. An increase in sympathetic activity has been reported in the following conditions: reduction of sleep duration over five nights, short sleep duration, low sleep efficiency and insomnia^59^. Different studies on sleep deprivation have found varying results.

Some studies have observed a decrease in sympathetic activity and an increase in parasympathetic activity, while another investigation did not show any significant alterations^59^. A low HRV was determined within a group of individuals with insomnia compared to good sleepers for the totality of sleep stages^60^. Nevertheless, in another investigation, no significant disparities were found between these two groups^61^.

A higher LF and LF/HF ratio was observed in a group of long-term nighttime shift nurses in comparison to their counterparts with daytime shift^62^. To ensure the accurate interpretation of HRV measurements results, it is recommended to have a consistent sleep routine prior to the day of measurement. Hence, documenting regular bedtime and wake-up time is of utmost importance for HRV investigations^63^.

Sleep quality is a critical determinant of HRV. Researchers should collect detailed sleep data, either through self-reports or objective measures like polysomnography or actigraphy, to account for its influence. Moreover, studies should consider excluding participants with sleep disorders or control for sleep quality in their analyses.

## Environmental factors

### Time of the day

HRV may be affected by the time of day the measurement is taken^64^. The circadian rhythm has a significant impact on several physiological functions, including the cardiovascular system^65^. Long-term measurement of HRV reported low HRV values during the late morning and early afternoon^64^. Healthy subjects have shown increased HRV during the night. It reached the acrophase during the second half of the night with the dominance of parasympathetic activity^65^.

Results from a trial measuring HRV at different times of the day showed significant differences between morning, afternoon and night results^11^. It was reported that the circadian rhythm of HR starts an upward trend upon awakening, or shortly after daily activities start^66^.

Generally, HRV parameters tend to increase at night and to decline during the day. However, a shift in HR, with the lowest values occurring at night and the highest values during the day was demonstrated in many studies^67–69^. Considering the previous observations, it is recommended to avoid combining data recordings collected at different times of the day. To minimize potential interference from intraday variability and to simplify the process for study participants, the advisable time to collect HR data is in the morning^11,64^.

### Temperature

HRV parameters are not under the exclusive control of the ANS and central nervous system, but they can also be affected by additional physiological factors, including body temperature regulation^26^. A significant drop in body temperature may lead to bradycardia, whereas an increase in body temperature may trigger tachycardia^26^.

An association between HRV and temperature was observed in individuals following their exposure to high-temperature, this was manifested by a reduction in HF parameter and an elevation in LF/HF ratio when the skin surface was heated^70^.

A study exploring HRV in a group of intensive care unit employees showed that physiological stress induced by temperature’s extremes (<36 °C and >39 °C) may lead to reduced HRV. This reduction can be induced by alterations in the regulatory process of the ANS^71^. In addition, studies have demonstrated correlations between HRV parameters and thermal stress^72,73^. In general, maintaining environmental temperature between 20 °C and 25 °C during HRV experiments is preferable^46,74^.

### Noises

Environmental noise can affect cardiovascular health through a stress mediated by the endocrine system and the ANS^75^. Studies reported that HRV can be susceptible to the effect of noise with variability dependent on type and level of noise^76–78^. Notably, low-frequency noise has been shown to have a negative impact on HRV^75^. A decline in LF values was observed in a group of individuals after exposure to speech noise, reflecting the alteration of sympathetic nervous system modulation^76^. However, it is recommended to measure HRV in a quiet environment to minimize the potential impact of external factors, such as noise, that can influence ANS balance and the accuracy of HRV measurement.

## Methodological factors

### Body position

Maintaining a consistent methodology for HRV data collection is crucial when considering the sensitivity of HRV measures to various physiological conditions, such as body posture adopted during data collection^11,64^. The effect of posture on autonomic outflow is well defined and correlates with a shift in the body axis. This response is predominantly sympathetic and occurs as a result of venous pooling which consequently involves the baroreflex^52^.

Several studies have highlighted the influence of different body positions on HRV measurement. In the lying position, there was a higher variation in many HRV parameters such as mean HR, SDNN, RMSSD, and pNN50(%) compared to the sitting position, and the LF component was higher in the sitting position than in the lying position^79^.

Rabbani et al. reported higher HRV values in the supine position, followed by declines during the sitting posture. Moreover, an additional reduction was observed in the standing position^80^.

Further studies confirmed increased HR values from the supine to the sitting position, and from the sitting to the standing position^38,81,82^. However, the body’s resting position can significantly influence the accurate quantification of HRV parameters^13^. Maintaining consistent body posture is an important consideration when monitoring HRV^64^. Furthermore, it has been observed that collecting data while seated is generally less complicated compared to data collection in a supine position^11^.

### Recording duration

The recording duration in HRV studies is determined by the specific nature of the investigation^9^. There are two groups of HRV analysis based on the duration of data recording; short-term HRV is commonly computed over 5 min, and long-term HRV is calculated most commonly over a 24-hour period^83^.

The Task Force of standards of HRV measurement recommended the use of 5 min short-term and 24 h long-term HRV recordings for time domain HRV analysis^9^. To compute low- and high-frequency components, a duration of at least 5 min is deemed suitable and recommended^84^. However, caution must be paid when evaluating very low frequency and ultra-low frequency from short-term recordings as their accurate analysis is only feasible when utilizing long term^84^.

More recently, researchers have proposed ultrashort-term HRV measurement (less than 5 min) as a more cost-effective alternative to the prohibitive cost of short-term recording ( ∼5 min), which has led to the limited applications of HRV measurement in medical care^85^. Numerous studies have indicated good agreement with the standard short-term recordings^86–88^.

A recent study by Vila et al. has shown that SDNN values obtained from 5-min recordings were higher than SDNN values obtained from the 1-minute segment^11^. Recording durations exceeding 2 min are unnecessary to obtain reliable RMSSD measures. Notably, even a standard ECG recording of 10-seconds can yield valid RMSSD values^19,86^.

Esco and Flatt reported that 1-min Ln-RMSSD (natural logarithm transform of RMSSD) provides better reliability compared to the conventional 5-min RMSSD^89^. Additional results concerning frequency domain HRV parameters were also reported. A minimum of 1-min continuous data collection is recommended by the Task Force guidelines for quantifying HF component^9^. Additional research has reported a significant agreement between 5-min measurement and ultrashort-term HRV recordings (<60 s)^89^.

Recent studies indicate the necessity of 1-min recordings for HF parameters, while 2-min recording duration is necessary for LF component^9,10^. It is important to recognize that for meaningful results and comparisons, researchers should exclusively conduct experiments with the same recording duration^11^. It is also advisable to reduce the duration of longer HRV recordings to align with the length of shorter durations^52^.

Another factor to consider when recording HRV is the acclimatization to the recording environment^52^. In most cases, HRV measurements are recorded without a standardized initial rest period to adjust the homoeostatic balance, hence the stationary prerequisite is altered^15^. Hence, starting HRV measurement with an acclimatization period which minimizes HRV variations caused by body position transitions can address this concern^52^. It is advisable not to inform participants about the measurement start, as their awareness may impact frequency respiration, potentially leading to alteration in ECG recordings^90^.

### Respiration

Respiration represents another significant factor that strongly influences HRV. HR increases during inspiration and decreases during expiration. This physiological phenomenon is known as respiratory sinus arrythmia (RSA)^26,91^. The regulation of respiration during HRV assessment remains a subject of ongoing debate among researchers. Controlling respiration may be recommended as respiratory depth and frequency can have an impact on HRV^19^. It has been suggested to maintain a respiratory frequency within the range of 9 to 24 cycles per minute, corresponding to the HF band (0.15–0.40 Hz). Consequently, any respiratory rate below or exceeding this HF band may compromise the accurate quantification of vagal tone^13,19^. However, the HF band could be adjusted according to the population of interest. For example, children and infants breathe rapidly, so it is recommended to shift the boundaries of the HF band to 0.24–1.04 Hz at rest^52^.

Some athletes have respiratory rates that are abnormally slow, which can hinder the conventional interpretation of the measured HF band^92^. Therefore, when selecting bands, it is crucial to consider the characteristics of the population, either by referring to previous research or by determining the respiratory rates of the data in question^19,52^. It is worth noting that frequency-domain HRV parameters reflecting vagal tone are more susceptible to respiratory effect compared to time-domain parameters (i.e.; RMSSD)^93^.

At present, controversial findings comparing spontaneous and paced breathing are reported. It was observed that controlling respiration could diminish respiratory effect on HRV^94^. Other trials demonstrated similar effects between controlled and spontaneous breathing^95^. Moreover, pacing respiration during HRV monitoring was suggested to alter the variability induced by the neural control of heartbeat, which may attenuate the variability that researchers are keen on studying^19^. Finally, multiple HRV trials miss reporting information on breathing, leading to difficulties in interpreting and comparing results.

## HRV measurement and emerging advancements

As discussed in this review, HRV is considered as a simple and valuable biomarker providing insights into the activity of a biological complex system- the autonomic nervous system interplay. However, there are challenges associated with the standardization, accuracy, and interpretability of HRV measurements. Recent advancements in technology and innovative methodologies aim to address these issues, thereby enhancing the reliability of HRV metrics across various contexts.

Wearable devices, such as smartwatches and fitness trackers, are at the forefront of HRV measurement advancements. These devices are equipped with sensors capable of continuously monitoring HRV in real-time. For instance, the integration of photoplethysmography and electrocardiography sensors in wearable devices allows for precise detection of R-R intervals^96^. Additionally, the capability to assess HRV continuously over extended periods enables the capture of diurnal variations, physical activity, and sleep patterns. Notably, long-term HRV monitoring can provide deeper insights into autonomic nervous system function^9,97^.

In tandem with sensor advancements, signal processing techniques have continuously evolved. Filtering and noise removal algorithms are critical for addressing issues related to HRV signal quality^98,99^.

Standardizing HRV measurement protocols is essential to obtain consistent and comparable results across studies. Although efforts are ongoing, many studies still fail to control for important factors discussed in this review. The aforementioned advancements can contribute to updating consensus guidelines, such as those from the Task Force of the European Society of Cardiology and the North American Society of Pacing and Electrophysiology^9,97^.

## Conclusion

HRV is a valuable, non-invasive indicator of ANS regulation of the cardiovascular system, made accessible by its ease-of-use and pain-free nature. However, the reliability and accuracy of HRV measurements are influenced by various physiological, lifestyle, environmental, and methodological factors, necessitating careful consideration (Table 1).

**Table 1:** Considerations for the assessment of heart rate variability.

**Influencing Factor**		**Recommendations for accurate measurement**
**Physiological factors**	Age	• The samples should be stratified based on age groups, or adjustments should be considered in statistical analyses.
	Gender	• The samples should be stratified based on sex groups, or adjustments should be considered in statistical analyses.
	Genetics	• Genetic factors are rarely introduced in HRV studies, for more accurate research results, they should be taken into account.
**Lifestyle factors**	Physical activity	• Given the effect of physical activity levels on HRV HRV, measurements should be conducted following, as the majority of studies suggest, 24 h after low-intensity aerobic exercise, 24–48 h after moderate-intensity exercise, and a minimum of 48 h after high-intensity exercise. • Moreover, the participants should be stratified based on baseline physical activity level, or adjustments should be considered in statistical analyses.
	Alcohol	• Alcohol should be avoided for a minimum 24 h prior to the assessment.
	Smoking	• Smoking should be avoided for a minimum10 h prior to the assessment.
	Drugs	• Cardiovascular-related medication administered by the subjects have to be controlled for these factors. • It is important to carefully conceptualize the inclusion or exclusion criteria, especially when considering health conditions and drugs use, in order to accurately analyze HRV data. This helps ensure that the data collected is relevant and reliable for the analysis.
	Caffeine	• Caffeine should be avoided for a minimum 2 h prior to the assessment.
	Food	• 2-hour period of food deprivation should be considered before measuring HRV. • Dietary data should be controlled and considered to ensure accurate HRV measurements.
	Sleep	• Consistent sleep routine prior to the day of HRV measurement should be maintained. • Document regular bedtime and wake-up time to ensure accurate HRV interpretation. • Consider excluding participants with sleep disorders or control for sleep quality in analyses.
**Environmental factors**	Time of the day	• Data recordings shouldn’t be performed at different times of the day to minimize potential interference from intraday variability.
	Temperature	• Temperature between 20 °C and 25 °C is preferred during HRV measurements.
	Noises	• Assessments should be performed in a quiet environment.
**Methodological factors**	Body position	• Body posture should be carefully chosen and maintained to avoid biases (especially for short term recordings). • Generally, setting position is preferred.
	Recording duration	• It is important to acclimatize participants to the recording environment before starting HRV measurements to stabilize homoeostatic balance. • It is preferable to not inform participants about the exact start of the measurement to prevent affecting respiratory frequency and altering ECG recordings.
	Respiration	• Considering the RSA phenomenon and its effects on HR, it is recommended to maintain the frequency within the range of 9 to 24 cycles per minute, especially when spectral analysis methods are used. Corresponding to the HF band (0.15–0.40 Hz) to reflects vagal tone.

Future research should focus on standardizing HRV measurement protocols, establishing population-specific reference norms, and integrating technology advancements. Advancements in signal processing techniques and comprehensive studies on the impacts of the influencing factors discussed in this review, and updating consensus guidelines are essential and will significantly enhance its application. Additionally, implementing educational programs for clinicians and researchers on the proper use and interpretation of HRV data will further improve its reliability and utility. Addressing these recommendations will strengthen HRV measurement, promoting better clinical and research outcomes in understanding and modulating autonomic nervous system dynamics.
